# Supplementary material for: Commercial influenza vaccines vary in HA-complex structure and in induction of cross-reactive HA antibodies
Source: Nat Commun. 2023 Mar 30;14:1763. doi: 10.1038/s41467-023-37162-z (PMC10060936; doi:10.1038/s41467-023-37162-z)
Supplement: Supplementary file 1 — Supplementary information [file 41467_2023_37162_MOESM1_ESM.pdf]

Supplementary Information for

Commercial influenza vaccines vary in HA-complex structure and in induction of cross-reactive HA antibodies

Mallory L. Myers[1*], John R. Gallagher[1*], Alexander J. Kim[1], Walker H. Payne[1], Samantha Maldonado-Puga[1], Haralabos Assimakopoulos[1], Kevin W. Bock[2], Udana Torian[1,+], Ian N. Moore[2,++] and Audray K. Harris [1#]

[1]Structural Informatics Unit, Laboratory of Infectious Diseases, National Institute of Allergy and Infectious Diseases, National Institutes of Health, 50 South Drive, Room 6351, Bethesda, MD, USA 20892

[2]Infectious Disease Pathogenesis Section, National Institute of Allergy and Infectious Diseases, National Institutes of Health, 33 North Drive, Room BN25, Bethesda, MD, USA 20892

+ Current Address: Laboratory of Human Carcinogenesis, National Cancer Institute, 37 Convent Drive, Room 306C, Bethesda, MD, USA 20892

++ Current Address: Yerkes National Primate Research Center, Emory University, 954 Gatewood Rd NE, Atlanta, GA, USA 30329 37

*co-authors, equal contribution

[#]To whom correspondence should be addressed:
Audray K. Harris
Email: harrisau@mail.nih.gov

This PDF file includes:

Supplementary Figure 1-11

a Vaccine Components

Name	Lot #	Total HA	HA/Strain	H1N1 Strain	H3N2 Strain	Influenza B-Victoria	Influenza B-Yamagata
Fluad	250792	45ug/0.5ml	15ug/0.5ml	A/Singapore/GP1908/2015 IVR-180	A/Singapore/INFIMH-16-2016 IVR-86	B/Maryland/15/2016	
Flublok	QFAA1816	180ug/0.5ml	45ug/0.5ml	A/Michigan/45/2015	A/Singapore/INFIMH-16-2016	B/Maryland/15/2016	B/Phuket/3073/2013
Fluzone HD	UJ000AA	180ug/0.5ml	60ug/0.5ml	A/Michigan/45/2015 x-275	A/Singapore/INFIMH-16-2016 IVR-86	B/Maryland/15/2016 BX-69A	
Flucelvax	252380	60ug/0.5ml	15ug/0.5ml	A/Singapore/GP1908/2015 IVR-180	A/NorthCarolina/04/2016	B/Iowa/06/2017	B/Singapore/INFTT-16-0610/2016

b Vaccine Manufacturing

Name	National Drug Code	Manufacturer	Vaccine Type	Valency	Inactivation	Method of Disruption	Reported Purification Method
Fluad	70461-018-04	Seqirus	Subunit, Adjuvanted	Trivalent	Formaldehyde	Cetyltrimethylammonium bromide	Zonal centrifugation
Flublok	49281-718-88	Sanofi Pasteur	Recombinant	Quadrivalent		Triton X-100	Column chromatography
Fluzone HD	49281-403-88	Sanofi Pasteur	Split Virus	Trivalent	Formaldehyde	Octyphenolethoxylate (Triton X-100)	Linear sucrose density gradient solution using continuous flow centrifuge
Flucelvax	70461-318-04	Seqirus	Subunit	Quadrivalent	b-propiolactone	Cetyltrimethylammonium bromide	"several process steps"

Supplementary Figure 1. Commercial influenza vaccines used in this study. Details are provided for the 4 commercial vaccines studied: Flucelvax, Flublok, Fluzone HD and Fluad. (a) The total HA concentration and the concentration of HA components such as H1 or H3 HA subtypes are denoted, as well as (b) production and inactivation methods.

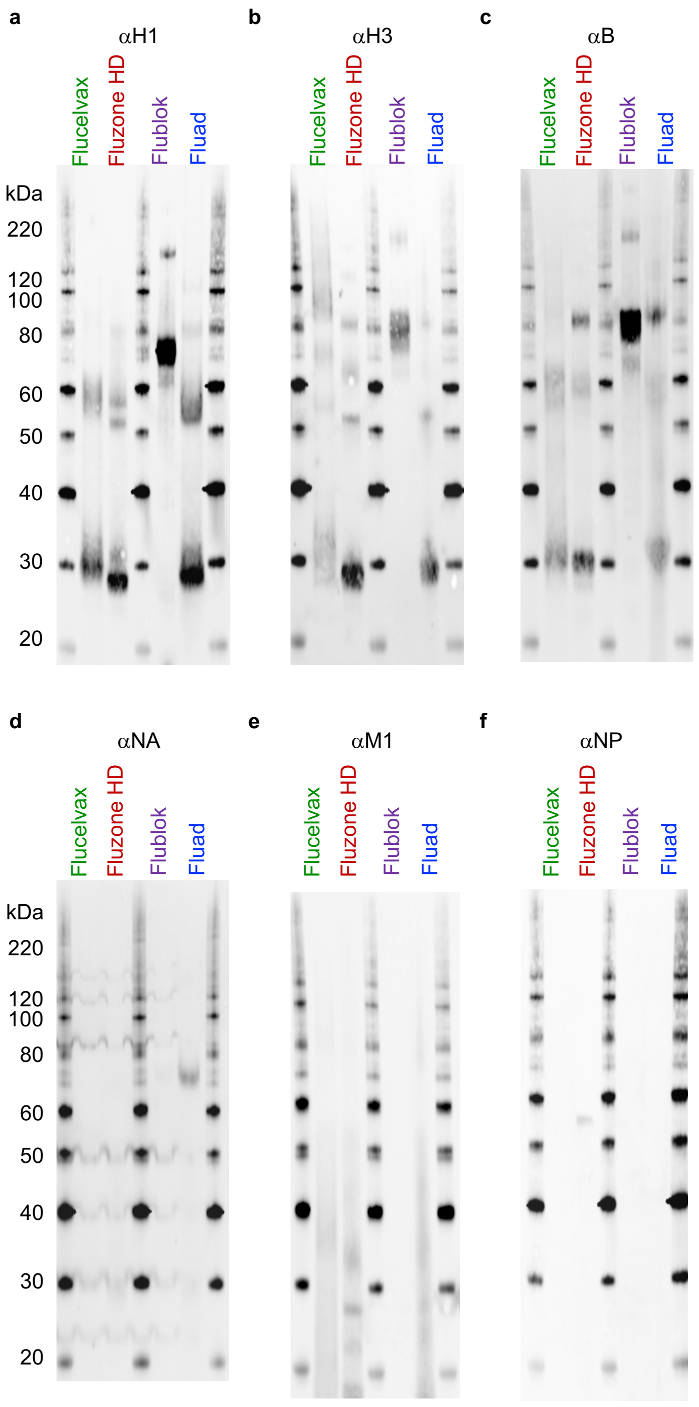

Supplementary Figure 2. Analyzing the composition of commercial influenza vaccines for influenza structural proteins HA, NA, M1 and NP. (a-f) Reactivity analysis via western blot of commercial influenza vaccines (Flucelvax, Fluzone HD, Flublok, Fluad) with primary antibodies to hemagglutinins (a) H1, (b) H3, and (c) influenza B. Also probed were (d) neuraminidase (NA), (e) matrix M1, and (f) nucleoprotein (NP). Molecular weight standards are given in panels *a* and *d*, and are the same for all panels. Blots were done in duplicate.

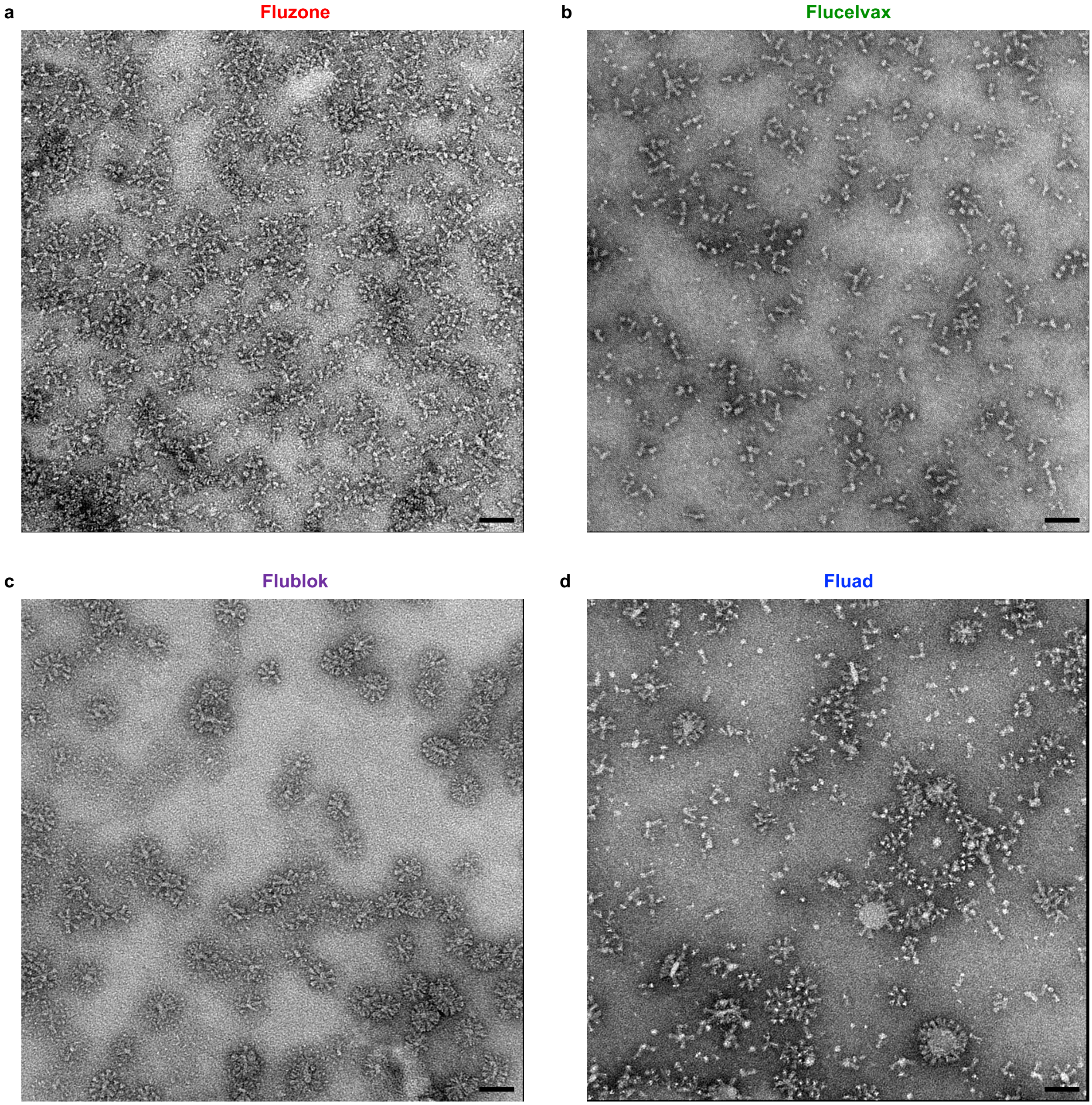

Supplementary Figure 3. Negative stain EM images of influenza vaccines.
Representative electron micrographs of the four influenza vaccines, illustrating the differential organization of HA proteins for (a) Fluzone HD, (b) Flucelvax, (c) Flublok, and (d) Fluad. Scale bar is 50 nm. Hundreds of micrographs of each of these vaccines have been collected.

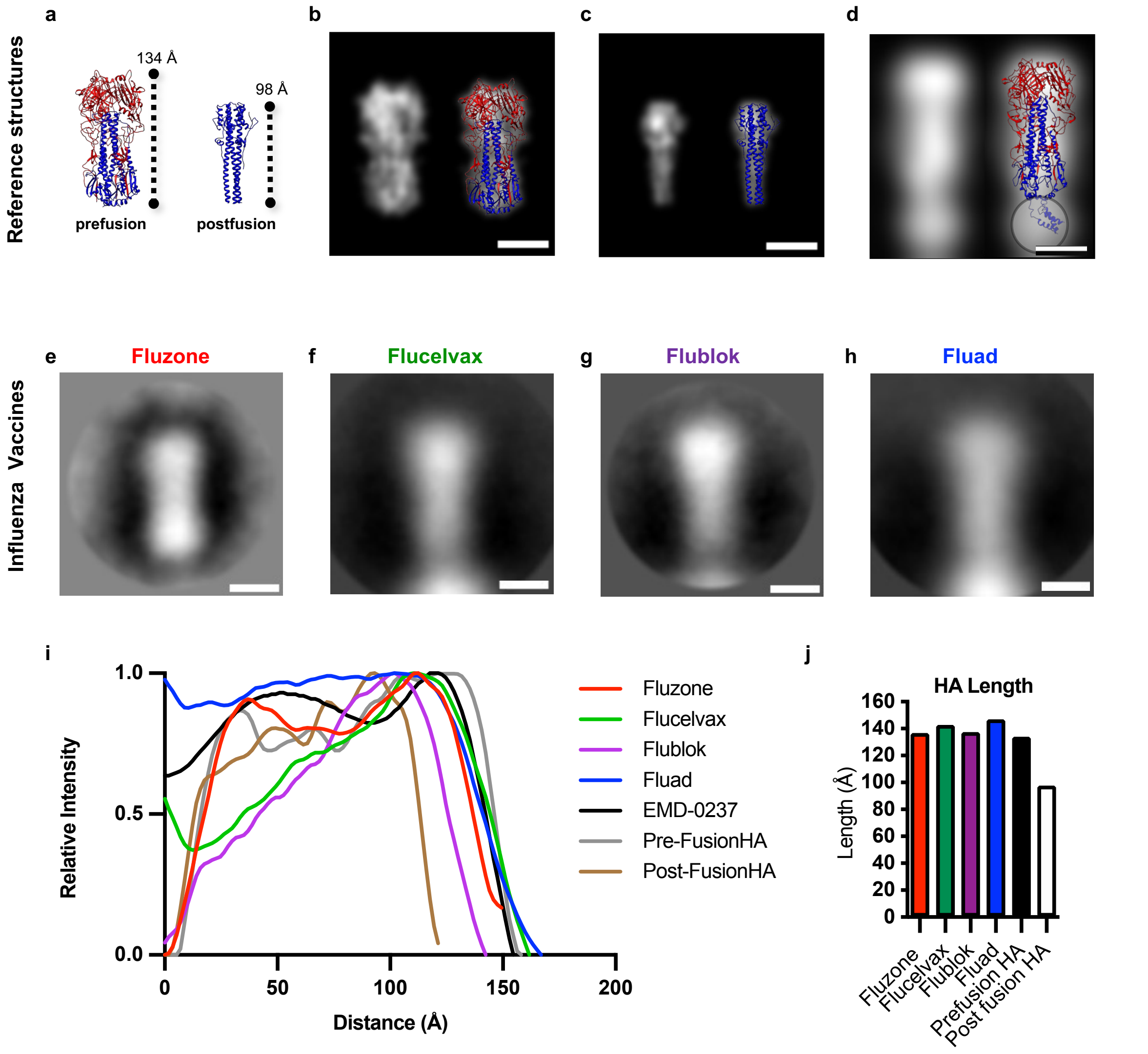

Supplementary Figure 4. Class averages of individual HA trimers in vaccines. (a) Ribbon-diagram depiction for prefusion (PDB ID 1HA0) postfusion (PDB ID 1HTM) HA structures. To compare x-ray crystallography-derived structures to EM images, PDB coordinates were used to calculate a volume with Chimera, then 2D projections were made with EMAN2 for (b) prefusion and (c) postfusion HA. (d) Reference structure of full-length HA in a lipid micelle, determined by single particle EM to 4.2 Å (EMD-0237) is given for comparison. (e-f) 2D-Class averages from negative-staining electron micrography manually picked, isolated HAs in vaccines (e) Fluzone, (f) Flucelvax, (g) Flublok, and (h) Fluad. (i) The HA density profile is plotted, where the positive x-axis traces along the threefold symmetry axis of HA trimers, starting from the base, continuing through the stem, then head, to the top of the HA trimer. (j) Total length of HA trimers, in the direction from the stem to the head. Scale bars are 50 Å.

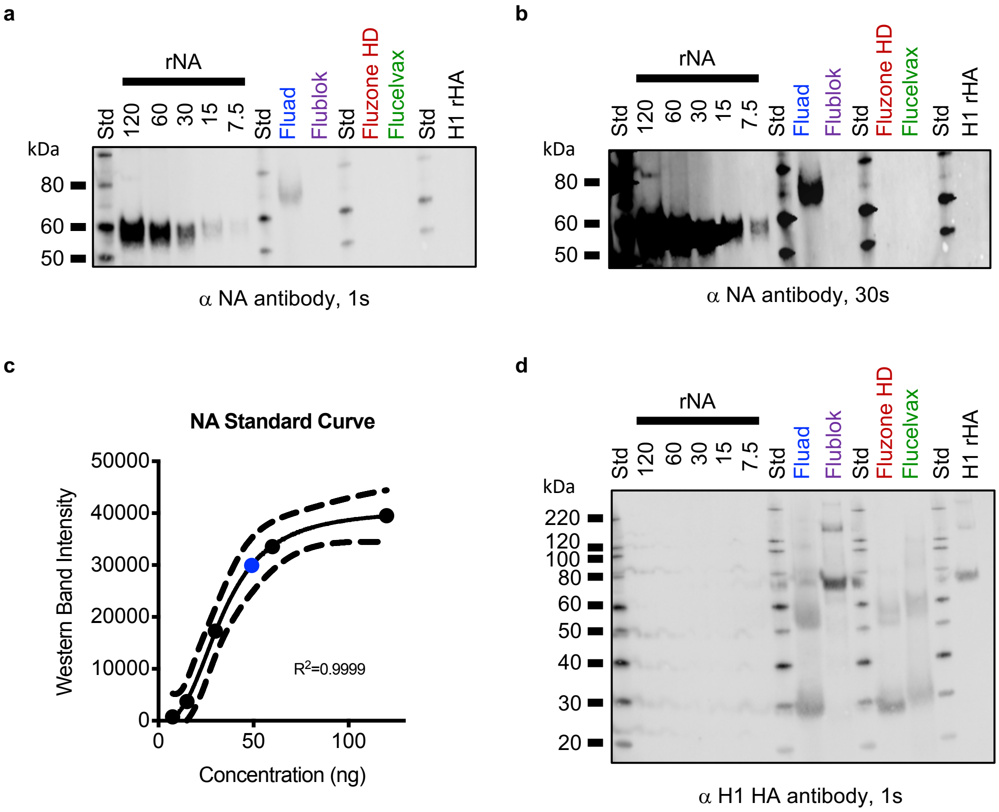

Supplementary Figure 5. Quantification of N1 NA in commercial vaccines by western blot. In panel *a-b*, decreasing concentrations of recombinant NA protein was applied (120, 60, 30,15, and 7.5 ng) in lanes 2-6. Remaining lanes were loaded with influenza vaccines and rHA at 0.3 ug H1 HA per sample. Blots were imaged for (a) 1 sec or (b) 30 sec. Uncropped blots in Source Data. A standard curve (black line) was generated for NA concentration (c) based on densitometry values from panel *a* (black dots) and shown with a 90% confidence interval (black dotted lines), the goodness of fit statistic (R^2) for the standard curve is also displayed. The concentration of NA in the Fluad vaccine was extrapolated from the curve (blue dot). A control blot (d) was probed with a polyclonal antibody to H1 HA and exposed for 1sec to ensure there was adequate protein transfer for detection. Focus was on N1 NA because H1N1 viruses caused pandemics in 1918 and 2009. Blots were done in duplicate.

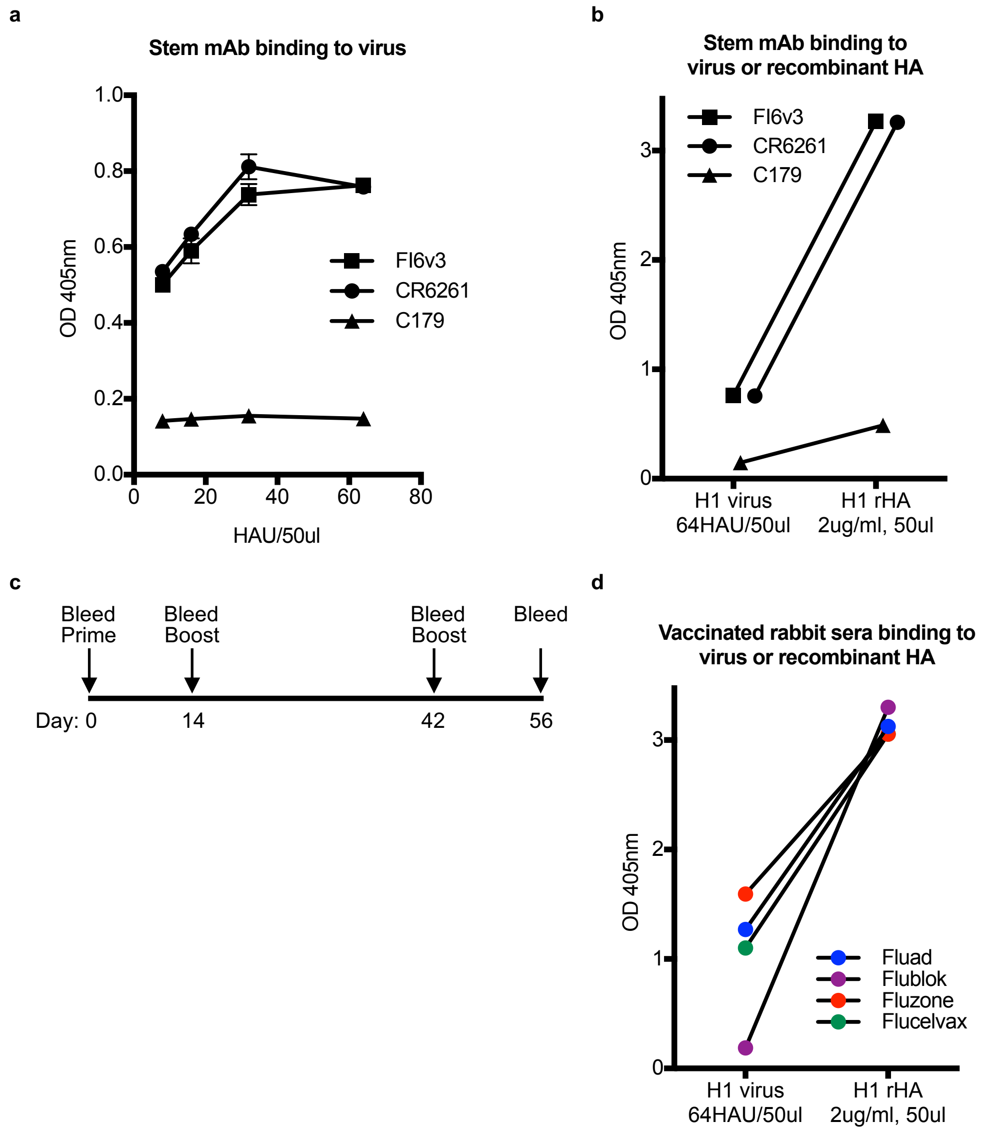

Supplementary Figure 6. Binding to HA displayed on virus. (a) Monoclonal stem antibodies (FI6v3 square, CR6261 circle, and C179 triangle) bind to increasing concentrations of virus. Trendline from N=2 replicates with standard error of the mean error bars shown. (b) The difference in binding on virus and rHA is shown for monoclonal stem antibodies from panel *a*. (c) Rabbit immunization with injections occurred on day 0, 14, and 42 and bleeds occurred on day 0, 14, 42, and 56. (d) The difference between binding to virus and rHA by immunized rabbit sera from day 56.

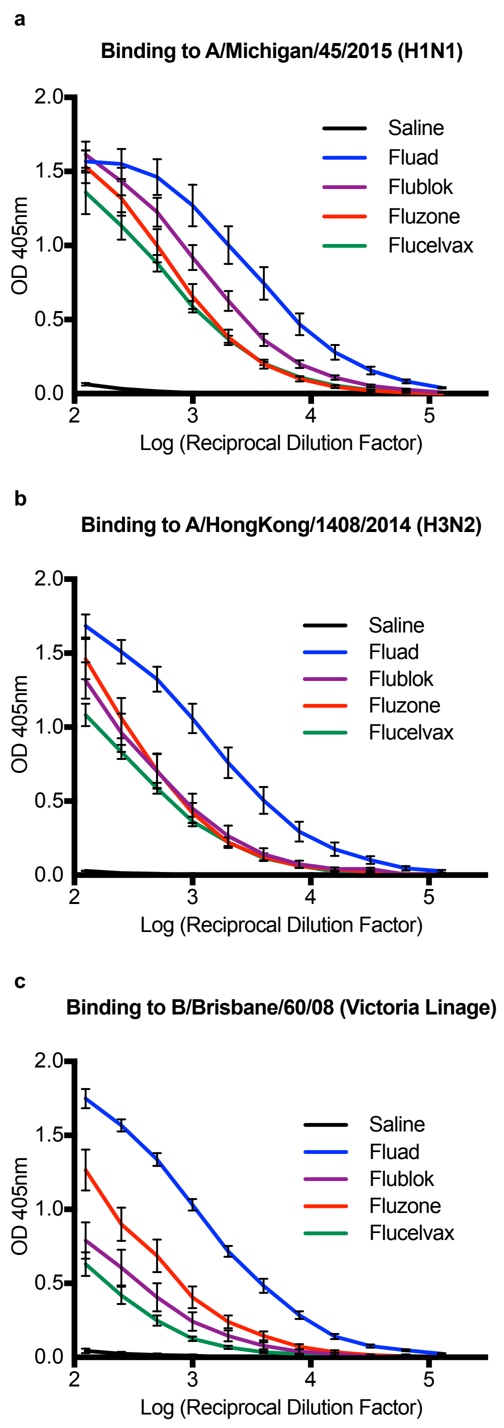

Supplementary Figure 7. Homosubtypic binding to hemagglutinin proteins from vaccinated mouse sera. Serially diluted sera from mice immunized with commercial vaccines Fluad, Flublok, Fluzone HD and Flucelvax were analyzed by ELISA for binding to antigenically matched HA proteins representing (a) H1N1, (b) H3N2, and (c) B-Victoria lineage influenza viruses. All graphs display mean (N=5 mice) with standard error of the mean error bars shown.

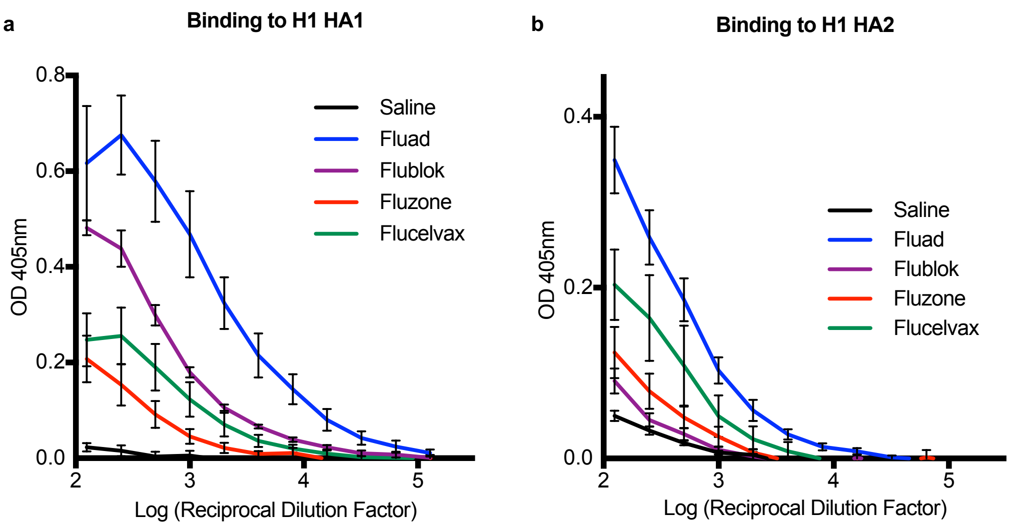

Supplementary Figure 8. HA head and stem epitope targeting of elicited antibodies from commercial influenza vaccines. Binding to recombinant protein representing the (a) recombinant H1 head and (b) recombinant headless H1 stem construct was compared by ELISA using serially diluted sera from mice immunized with commercial vaccines Fluad, Flublok, Fluzone HD, Flucelvax, and with saline as a control. Graphs display mean (N=15 mice) with standard error of the mean bars. Curves were used to derive endpoint titers shown in main-text figure 5.

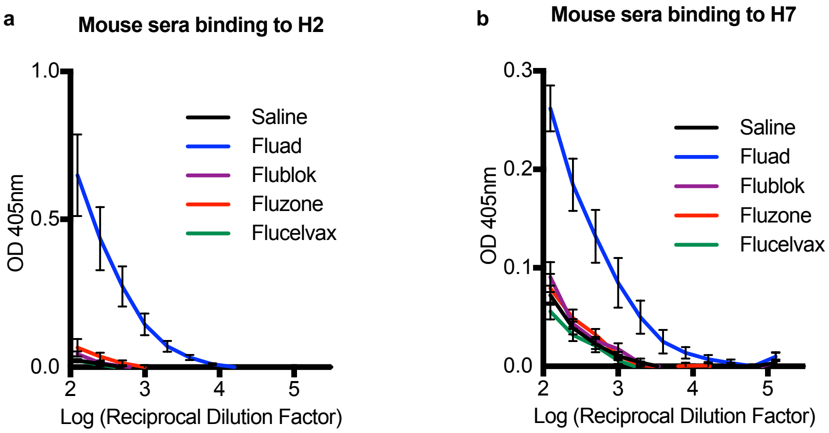

Supplementary Figure 9. Cross-reactive immunogenicity of vaccinated mouse sera for H2 and H7 HA. Binding to full-length (a) H2 and (b) H7 proteins were compared by ELISA of serially diluted sera from mice immunized with commercial vaccines Fluad, Flublok, Fluzone HD, Flucelvax, and with saline as a control. Graphs display mean (N=5 mice) with standard error of the mean bars. Curves were used to derive endpoint titers shown in main-text figure 5.

a

Mouse IDs/ Vaccine immunization condition

ID	Condition	Day
671	BSS BSA	3
672	BSS BSA	3
646	Saline	3
647	Saline	3
651	Flucelvax	3
652	Flucelvax	3
656	Fluzone HD	3
657	Fluzone HD	3
661	Flublok	3
662	Flublok	3
666	Fluad	3
667	Fluad	3
673	BSS BSA	6
674	BSS BSA	6
648	Saline	6
649	Saline	6
653	Flucelvax	6
654	Flucelvax	6
658	Fluzone HD	6
659	Fluzone HD	6
663	Flublok	6
664	Flublok	6
668	Fluad	6
669	Fluad	6
675	BSS BSA	14
655	Flucelvax	14
660	Fluzone HD	14
665	Flublok	14
670	Fluad	14

b

Blinded evaluation of lung pathology

Viral Staining	TCL
Neg	Neg
Neg	Neg
Interstitial	Med
Interstitial	High
Airway	Low
Airway	Low
Airway	Low
Airway	Low
Neg	Neg
Airway	Low
Neg	Neg
Neg	Neg
Neg	Neg
Neg	Neg
Interstitial	Med
Interstitial	High
Airway	Low
Airway	Low
Airway	Low
Airway	Med
Neg	Neg
Airway	Low
Neg	Neg
Neg	Neg
Neg	Neg
Airway	Low
Airway	Low
Airway	Low
Airway	Low

c

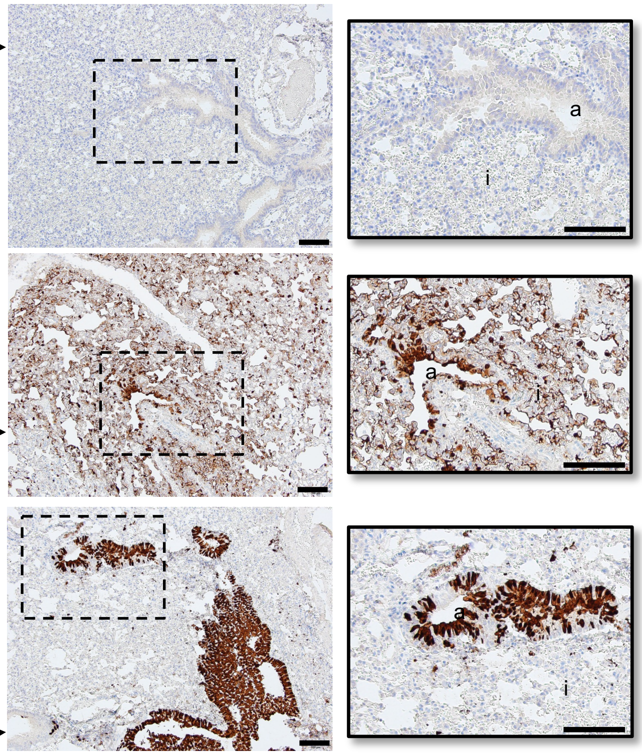

Supplementary Figure 10. Analysis approach to histopathology after H1N1 challenge. An unbiased, blinded method was employed for analysis of histopathology of mouse lungs following H1N1 challenge. (a) Mouse identification numbers (IDs) were generated to blind the type of immunogen and challenge given. (b) Blinded evaluation of tissue samples was used to classify viral presences as negative, low, medium, and high. (c) Selected sample images illustrate immunohistochemistry staining (IHC) against the influenza nucleoprotein protein for groups corresponding to negative healthy lung, interstitial antigen staining, and airway antigen staining. Within panel *c,* letter abbreviations are: a = airway, i = pulmonary interstitium. Scale bars are 100 µm. Pathology results were replicated with 2 mice per condition.

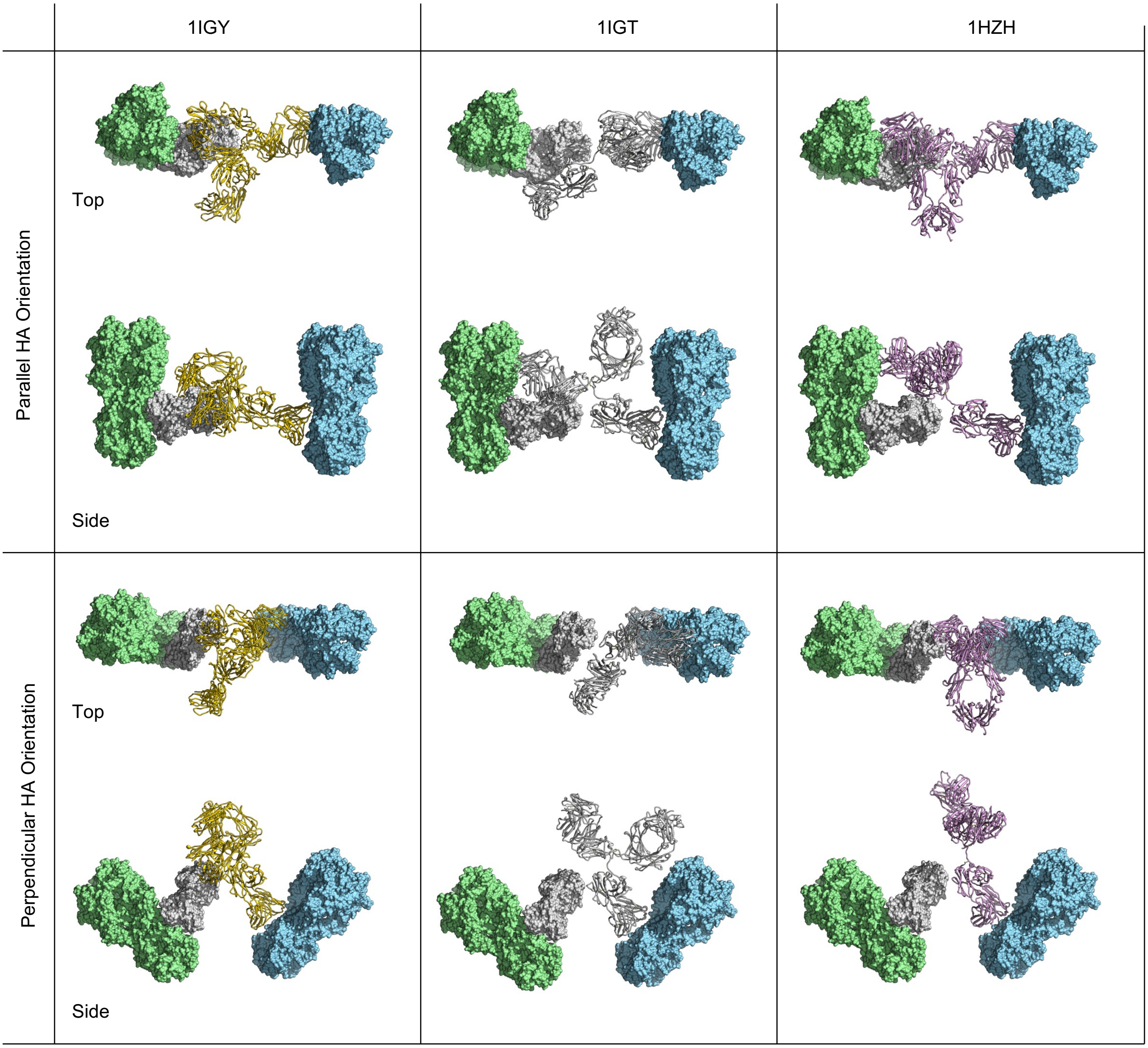

Supplementary Figure 11. Visualization of full-length IgG binding steric constraints. HA trimers (green and blue) modeled in close proximity but with enough space to permit Fab domain binding are illustrated for HA trimers that are parallel (top), or perpendicular (bottom). Three different PDB structures of full-length IgG are illustrated in each of the three columns (ribbon representation). Bound Fab for a second IgG is illustrated in gray space filling representation. The bases of the parallel HA molecules are 185 Å apart, and the bases of perpendicular HA molecules are 80 Å apart. Considering the flexibility available to the second IgG, the conformations given by PDB IDs 1IGT and 1HZH on perpendicular HA trimers are capable of avoiding steric clashes with a second IgG, while the conformation given by 1IGY is likely to be in steric clash. For parallel HA trimers, all three IgG conformations illustrated are likely to be in steric clash with a second IgG binding event.
