# Supplementary material for: Commercial influenza vaccines vary in HA-complex structure and in induction of cross-reactive HA antibodies
Source: Nat Commun. 2023 Mar 30;14:1763. doi: 10.1038/s41467-023-37162-z (PMC10060936; doi:10.1038/s41467-023-37162-z)
Supplement: Supplementary file 2 — Reporting Summary [file 41467_2023_37162_MOESM2_ESM.pdf]

nature portfolio

Corresponding author(s): Audray K. Harris

Last updated by author(s): Mar 2, 2023

Reporting Summary

Nature Portfolio wishes to improve the reproducibility of the work that we publish. This form provides structure for consistency and transparency in reporting. For further information on Nature Portfolio policies, see our Editorial Policies and the Editorial Policy Checklist.

Statistics

For all statistical analyses, confirm that the following items are present in the figure legend, table legend, main text, or Methods section.

n/a	Confirmed	
☐	☒	The exact sample size (*n*) for each experimental group/condition, given as a discrete number and unit of measurement
☐	☒	A statement on whether measurements were taken from distinct samples or whether the same sample was measured repeatedly
☐	☒	The statistical test(s) used AND whether they are one- or two-sided<br>*Only common tests should be described solely by name; describe more complex techniques in the Methods section.*
☒	☐	A description of all covariates tested
☒	☐	A description of any assumptions or corrections, such as tests of normality and adjustment for multiple comparisons
☐	☒	A full description of the statistical parameters including central tendency (e.g. means) or other basic estimates (e.g. regression coefficient) AND variation (e.g. standard deviation) or associated estimates of uncertainty (e.g. confidence intervals)
☐	☒	For null hypothesis testing, the test statistic (e.g. *F*, *t*, *r*) with confidence intervals, effect sizes, degrees of freedom and *P* value noted<br>*Give P values as exact values whenever suitable.*
☐	☒	For Bayesian analysis, information on the choice of priors and Markov chain Monte Carlo settings
☒	☐	For hierarchical and complex designs, identification of the appropriate level for tests and full reporting of outcomes
☒	☐	Estimates of effect sizes (e.g. Cohen's *d*, Pearson's *r*), indicating how they were calculated

Our web collection on statistics for biologists contains articles on many of the points above.

Software and code

Policy information about availability of computer code

Data collection	SerialEM 3.6
Data analysis	SerialEM 3.6, RELION3 3.1.2 git pull 4/23/2021, RELION 2.1 git pull 1/8/2018, CTFfind 4.1.10, Chimera 1.16, MolProbability Probe 2.16.130520, IMOD 4.10.16, EMAN2 2.91, R 4.1.3, Prism 9.3.1, FIJI 2.3.0, PERL v5.30.3

For manuscripts utilizing custom algorithms or software that are central to the research but not yet described in published literature, software must be made available to editors and reviewers. We strongly encourage code deposition in a community repository (e.g. GitHub). See the Nature Portfolio guidelines for submitting code & software for further information.

Data

Policy information about availability of data

All manuscripts must include a data availability statement. This statement should provide the following information, where applicable:

- Accession codes, unique identifiers, or web links for publicly available datasets
- A description of any restrictions on data availability
- For clinical datasets or third party data, please ensure that the statement adheres to our policy

The source data underlying Figs. 2a, 3j, and 4g, 5a,c,d,e,f and 6b,c,d,e and Supplementary Figs. 4i,j, 5c, and 6a,b,d are provided as SourceData.xlsx inside SourceData.zip. Tomographic reconstructions have been deposited in the Electron Microscopy Data Bank, https://www.ebi.ac.uk/emdb/, under the following accession numbers: Fluad spiked nanodisc, EMDB-27232; Flublok starfish, EMD-27233. PDB files used in this study were downloaded from the Protein Data Bank: https://www.rcsb.org/, including 1HZH, 1IGT, 1IGY, 5DK3, 6GFE, 4HLZ. Code to reproduce Fig. 2a is available inside SourceData.zip

Human research participants

Policy information about studies involving human research participants and Sex and Gender in Research.

Reporting on sex and gender	not applicable
Population characteristics	not applicable
Recruitment	not applicable
Ethics oversight	not applicable

Note that full information on the approval of the study protocol must also be provided in the manuscript.

Field-specific reporting

Please select the one below that is the best fit for your research. If you are not sure, read the appropriate sections before making your selection.

☒ Life sciences ☐ Behavioural & social sciences ☐ Ecological, evolutionary & environmental sciences

For a reference copy of the document with all sections, see nature.com/documents/nr-reporting-summary-flat.pdf

Life sciences study design

All studies must disclose on these points even when the disclosure is negative.

Sample size	Sizes of animal groups were minimized for ethical concerns but still provide statistical significance. Since the effect size of the immune response following vaccination was unknown a power analysis was inappropriate. Since one way ANOVA was going to be the statistical test outcome measure, determining appropriate degrees of freedom was possible and utilizing the resource equation method was a suitable alternative. Based upon the resource equation method, the degree of freedom for the final ANOVA should be a minimum of degrees of freedom/number of groups+1 (10/5+1=3) and a maximum of degrees of freedom/number of groups+1 (20/5+1=5), so 5 mice per group were selected for mouse challenge studies and 4 were selected for pathology. Arifin WN, Zahiruddin WM. Sample size calculation in animal studies using resource equation approach. Malays J Med Sci. 2017;24(5):101–105. https://doi.org/10.21315/mjms2017.24.5.11<br>1-2 rabbits were vaccinated per vaccine type for sera generation due to limitation based upon the amount of protein necessary for their multiple immunization schedule.
Data exclusions	No data were excluded from the analyses.
Replication	The biological replicates were stated in each figure legend (n=). The mouse lethal challenge experiment was replicated twice with earlier endpoints for tissue collection, the bodyweight data for the first 3 days following challenge was consistent between the three experiments. Generation of immunized rabbit sera was conducted once, however prior to termination rabbit sera was tested and confirmed production of antibodies consistent to those from previous mouse immunization experiments.
Randomization	For all experiments animals (mice or rabbits) were randomly assigned to treatment groups either: vaccine, saline, or uninfected control groups.
Blinding	An unbiased blinded then unblinded approach was used to analyze data collected from mouse lung tissue: histopathology and TCID50. Mouse daily observations following challenge were conducted blindly, however mouse daily weights were collected by an unblinded experimenter due to pragmatic reasons. Rabbit immunizations experiments were done blind to experimental group. All of the serological assays including ELISA, western, tiering, and structural, biochemical and biophysical characterization of the antibodies were not performed in a blinded manner.

Reporting for specific materials, systems and methods

We require information from authors about some types of materials, experimental systems and methods used in many studies. Here, indicate whether each material, system or method listed is relevant to your study. If you are not sure if a list item applies to your research, read the appropriate section before selecting a response.

Materials & experimental systems

n/a	Involved in the study
☐	☒ Antibodies
☐	☒ Eukaryotic cell lines
☒	☐ Palaeontology and archaeology
☐	☒ Animals and other organisms
☒	☐ Clinical data
☐	☒ Dual use research of concern

Methods

n/a	Involved in the study
☒	☐ ChIP-seq
☒	☐ Flow cytometry
☒	☐ MRI-based neuroimaging

Antibodies

Antibodies used	C179, FI6v3, CR6261 were provided for use by The Dale and Betty Bumpers Vaccine Research Center (VRC) at the National Institutes of Health (NIH). C179, FI6v3, CR6261 were applied to microtiter plates (immulon 2 B, Thermo Fisher) at 1.25 μg/ml. Anti-NA antibody ThermoFisher Scientific Influenza A H1N1 NA Polyclonal Antibody PA5-32238 was used a 1:1000 dilution. Anti-H1 polyclonal PS6000 (Protein Sciences) was used at 1:1000 dilution. Anti-H3 polyclonal PS6001 (Protein Sciences) was used at 1:1000 dilution. Anti-HB polyclonal PS6003 (Protein Sciences) was used at 1:1000 dilution. Anti-M1 monoclonal HB64 (American Type Culture Collection clone M2-1C6-4R3) was used at 3.5 μg/ml. Anti-NP monoclonal HB65 (American Type Culture Collection clone H16-L10-4R5) was used at 3.5 μg/ml.
Validation	Antibody C179 (mouse) was first described in PubMed ID 7682624. FI6v3 (human) was first described in PubMed ID 21798894. CR6261 (human) was first described in PubMed ID 19079604. Protein Sciences antibodies were validated for western and ELISA applications. HB64 is described in 78 citrations at ATCC, and HB65 is described in 392 citations at ATCC. No additional validation was conducted prior to experimentation.

Eukaryotic cell lines

Policy information about cell lines and Sex and Gender in Research

Cell line source(s)	Madin-Darby Canine Kidney (MDCK-ATL) cells were obtained from International Reagent Resource (IRR).
Authentication	The cell lines were not authenticated.
Mycoplasma contamination	Cell lines were not tested for mycoplasma contamination.
Commonly misidentified lines (See ICLAC register)	We did not use any commonly misidentified lines.

Animals and other research organisms

Policy information about studies involving animals; ARRIVE guidelines recommended for reporting animal research, and Sex and Gender in Research

Laboratory animals	Female BALB/c mice (Taconic Biosciences) aged 8-10 weeks at experimental start were utilized in these experiments. Mice were maintained in a facility on a 14 hours light and 10 hours dark cycle at an ambient temperature of 22 ± 3 °C, and a humidity between 30-70%, recorded daily. New Zealand White rabbits (Cocalico Biologicals) began experimentation at approximately two to four months of age.
Wild animals	The study did not involve wild animals.
Reporting on sex	Female mice were used for all experiments based upon the higher immunogenetic response following vaccination. Fink AL, Engle K, Ursin RL, Tang WY, Klein SL. Biological sex affects vaccine efficacy and protection against influenza in mice. Proc Natl Acad Sci USA. 2018;115(49):12477-82. Male rabbits were utilized and displayed a similar immune profile to the mouse studies.
Field-collected samples	The study did not involve samples collected from the field.
Ethics oversight	All mouse experiments were preformed under protocols approved by the Animal Care and Use Committee (ACUC) at the National Institute of Allergy and Infectious Disease (NIAID). The studies were performed in accordance with all federal regulations, NIH guidelines, AAALAC, and IACUC approval. Rabbit studies were conducted by Life Sciences Solutions (Thermo Fisher Scientific) and were in accordance with the ACUC of Cocalico Biologicals, Inc.

Note that full information on the approval of the study protocol must also be provided in the manuscript.

Dual use research of concern

Policy information about dual use research of concern

Hazards

Could the accidental, deliberate or reckless misuse of agents or technologies generated in the work, or the application of information presented in the manuscript, pose a threat to:

No	Yes	
☒	☐	Public health
☒	☐	National security
☒	☐	Crops and/or livestock
☒	☐	Ecosystems
☒	☐	Any other significant area

Experiments of concern

Does the work involve any of these experiments of concern:

No	Yes	
☒	☐	Demonstrate how to render a vaccine ineffective
☒	☐	Confer resistance to therapeutically useful antibiotics or antiviral agents
☒	☐	Enhance the virulence of a pathogen or render a nonpathogen virulent
☒	☐	Increase transmissibility of a pathogen
☒	☐	Alter the host range of a pathogen
☒	☐	Enable evasion of diagnostic/detection modalities
☒	☐	Enable the weaponization of a biological agent or toxin
☒	☐	Any other potentially harmful combination of experiments and agents
