# Supplementary material for: Commercial influenza vaccines vary in HA-complex structure and in induction of cross-reactive HA antibodies
Source: Nat Commun. 2023 Mar 30;14:1763. doi: 10.1038/s41467-023-37162-z (PMC10060936; doi:10.1038/s41467-023-37162-z)
Supplement: Supplementary file 3 — Description of additional supplementary files [file 41467_2023_37162_MOESM3_ESM.pdf]

Description of additional supplementary files for:

Commercial influenza vaccines vary in HA-complex structure and in induction of cross-reactive HA antibodies

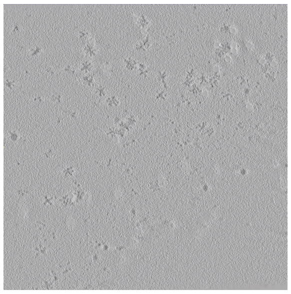

Supplementary Movie 1. Slicing through a 3D tomogram of the commercial influenza virus vaccine Flublok. HA complexes appear as starfish structures. Within each complex, HA trimers emanate outward from a central focus in all directions.

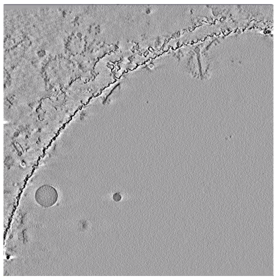

Supplementary Movie 2. Slicing through a 3D tomogram of the commercial influenza virus vaccine Fluad. Two Fluad spiked nanodiscs are shown proximal to the carbon edge of the hole on the EM grid. As the visualization plane slices through the sample, a dark band of density transits from one side of the disc to the other, which is the result of the oblique angle of the complex to the imaging plane. One Fluad spiked nanodisc is in the upper right, while another Fluad spiked nanodisc is to the left. An example of an adjuvant vesicle is in the lower left and appears as an approximate grey sphere.

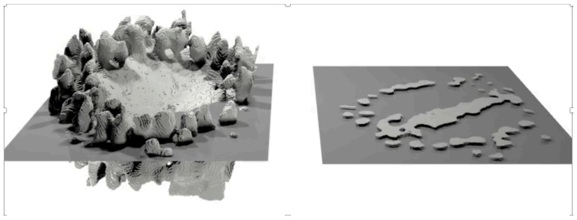

Supplementary Movie 3. Fluad spiked nanodisc from cryo-electron tomography viewed as a 3D solid surface rendered as slices of the volume. (Left) Successive plane slicing through a solid 3D surface rendering of the 3D tomographic volume of a Fluad complex (i.e. spiked nanodisc). (Right) Corresponding planes only showing the density in the plane of the slice. The glycoprotein spikes of the complex appear as dotted densities around a central band of membrane.
